# Is It Beneficial to Patients to Include Congenital Adrenal Hyperplasia (CAH) Among the Disorders of Sex Development (DSD)?

**DOI:** 10.3389/fped.2018.00344

**Published:** 2018-11-12

**Authors:** Ricardo González, Barbara M. Ludwikowski

**Affiliations:** Pediatric Surgery and Urology, Kinder-und Jugendkrankenhaus AUF DER BULT, Hanover, Germany

**Keywords:** congenital adrenal hyperplasia (CAH), disorder of sex development (DSD), feminizing genitoplasty, urogenital sinus mobilization, gender identidy

Congenital adrenal hyperplasia (CAH) is the most common cause of ambiguous genitalia in genetic females. It results from enzyme deficiencies involved in the synthesis of corticosteroids which cause the accumulation of steroid precursors with androgenic effect. When this defect is present in 46 XX individuals, in addition to the pathological effects resulting from the deficiency in gluco- and mineralocorticoids, fetal virilization occurs. Prompt diagnosis and treatment after birth can be lifesaving. Since the condition is hereditary, prenatal diagnosis and treatment is possible and results in decrease virilization (1).

Girls with CAH who are diagnosed and treated with steroid replacement since birth and raised in the female gender and receive adequate endocrinological management and follow up have the potential for fertility and adequate sexual function in adulthood with a low risk for later gender dysphoria (2, 3). Of course, corrective surgery to create separate external openings for the urethra and the vagina is often necessary to achieve these goals. The timing of corrective surgery is controversial but should be before puberty to avoid problems with menstruation than can lead to hydro-hematocolpos, hydro-hematometra, endometriosis, and even sepsis (4). Whether or when an operation to reduce the size of the usually enlarged clitoris ought to be done is a matter of debate but the current trend is to wait until the girl can participate in the decision.

The term disorders (or differences) of sex development (DSD) was coined in 2006 at a consensus meeting in Chicago to replace such terms as intersex, pseudo hermaphroditism, etc., which were considered pejorative by many affected individuals (5). Although the term DSD was widely accepted by patients, families and health professionals, it created a new series of problems, partly because the conditions included under this umbrella term was made in an arbitrary fashion. An important topic of debate in this area is the gender assignment of a newborn with ambiguous genitals. Although in some cases there is a dilemma stemming from the uncertain natural history and prognosis of a given condition (such as is often the case in patients with asymmetrical gonadal dysgenesis, ovotesticular DSD, or partial androgen insensitivity), other conditions included under the DSD umbrella present no such dilemma, such as proximal hypospadias with scrotal testes or complete androgen insensitivity since the genital appearance can easily identified as male or female.

Some patient advocacy groups, which often include affected individuals dissatisfied with their medical experiences, were instrumental in proposing new guidelines for the diagnosis and treatment of DSD. This sometimes resulted in proposals of dubious rationality, such as a moratorium on all surgical procedures on the genitals in children without life threatening conditions (6) and influencing recommendations by human rights groups and legislatures. The arbitrary nature of this recommendation is best demonstrated by the exclusion of non-medical circumcision or orchidopexy from this moratorium. Also the often proposed recommendation of postponing gender assignment until a later age (a practice which is now legal in some countries) lacks evidence to support its potential benefits and long term consequences for the mental health of affected individuals are unknown. Much remains to be learned about the optimal management of many of these conditions and good quality, prospective studies are necessary to move the arguments from mere opinions and anecdotes to scientific evidence.

When the above mentioned attitudes are applied to conditions like hypospadias or CAH, it is doubtful that in the long term they will be beneficial to patients. The long term outcomes of proximal hypospadias in males and CAH in females are well-documented. Despite the imperfect long term results of current therapy for these conditions, improvement of available medical, surgical and treatments should lead to improved outcomes in the future, as has been the case in the last three decades. To abandon the present methods of management of these conditions to replace them with new unproven protocols with unknown outcomes seems irresponsible.

Therefore, we and others have questioned the inclusion of CAH from the umbrella term DSD (7–9). Such exclusion may be also beneficial for other conditions. We propose that the use of the name of specific diagnosis for each condition avoiding lumping many diverse diagnosis together a practice that leads to generalizations and oversimplifications and is ultimately not helpful to patients and families.

In cases of 46 XX newborns with CAH, this would make female gender assignment at birth unquestionable. Surgical correction should take place in the first year of life to separate the openings of the urethra and vagina to normalize urinary and genital functions (10–12), in as is done currently for repair of hypospadias in boys. It is our practice to conceal the hypertrophied clitoris with a hood and to postpone clitoral reduction if needed until the patient is able to participate in the decision. The practice of reducing the clitoris early in infancy and postponing correction of the urogenital sinus until after puberty is to be condemned.

## Author contributions

All authors listed have made a substantial, direct and intellectual contribution to the work, and approved it for publication.

### Conflict of interest statement

The authors declare that the research was conducted in the absence of any commercial or financial relationships that could be construed as a potential conflict of interest.
